# Type 1A endoleak detachable coil embolization after endovascular aneurysm sealing: Case report

**DOI:** 10.1016/j.ijscr.2021.106024

**Published:** 2021-05-26

**Authors:** E. Dinoto, F. Ferlito, D. Mirabella, G. Tortomasi, G. Bajardi, F. Pecoraro

**Affiliations:** a Vascular Surgery Unit, AOUP Policlinico ‘P. Giaccone’, Palermo, Italy; b Department of Surgical, Oncological and Oral Sciences, University of Palermo, Italy

**Keywords:** Endoleak, Nellix, Coil embolization, Aneurysm

## Abstract

**Introduction:**

Endovascular aneurysm sealing (EVAS) with the Nellix system was introduced to reduce endovascular aneurysm repair (EVAR) perioperative complications, especially endoleaks. Herein we report a case of successful type 1A endoleak managed with detachable coils embolization after EVAS.

**Presentation of case:**

A 77-year-old male was referred for abdominal pain. The angio-CT scan confirmed the previous EVAS procedure and showed a type Is2 endoleak below the right renal artery resulting in a 2.5 cm aortic blister with contrast medium filling the space between the aortic wall and the endobags. The patient was considered unfit for conventional open surgery and an endovascular approach with coil embolization Concerto Helix Detachable Coil System was chosen under local anesthesia. After intervention, a complete abdominal pain regression was registered. The 12- month CT follow-up showed endoleak sealing and Nellix system stability.

**Discussion:**

EVAS has been associated to a high endoleaks and complications incidence when compared to EVAR. The EVAS different device concept led to a different endoleak classification and management. Endoleak management main options include the Nellix system explantation or the Nellix in Nellix application, however these are nearly always not applicable, respectively, due to the high surgical risk condition and the Nellix system availability, especially in emergent setting. Despite the use of coil embolization is controversial, this tool is off-the-shelf and leads to a disease resolution in most of patients without other surgical options.

**Conclusion:**

Proximal type Is2 embolization after EVAS is feasible with limited invasiveness.

## Highlights

• Endovascular aneurysm sealing with the Nellix system was introduced to reduce perioperative complications;
• EVAS has been associated to a high endoleaks and complications incidence when compared to EVAR;
• The use of coil embolization is controversial, this tool can be an alternative in patients without other surgical options;
• Proximal endoleak type I embolization after EVAS is feasible with limited invasiveness

## 1 Introduction

Endovascular repair of aortic aneurysm (EVAR) is a continuously increasing procedure as it represents the gold standard in abdominal aortic aneurysm (AAA) treatment. Endovascular aneurysm sealing (EVAS), with the Nellix system (Endologix Inc., Irvine, CA, US) has been introduced with the aim to reduce EVAR perioperative complications, especially endoleaks by filling endobags in order to seal the aneurysm [1]. Despite encouraging short outcomes, mid- and longer-term results have shown a significant higher incidence of EVAS related complications, and in 2019 the Nellix system (Endologix Inc.) has been withdrawn from the marked [2]. Herein we report a case of type 1A endoleak after EVAS managed with detachable coils embolization.

This work has been written in accordance with the SCARE criteria [3].

## 2 Case report

A 77-year-old male with hypertension, diabetes mellitus, reduced cardiac output (35% cardiac ejection fraction), moderate renal failure was referred for abdominal pain. At admission, his physical examination revealed regular heart rate of 80 beats/min, blood pressure of 140/70 mmHg and temperature of 36.8 °C. At history, he referred a previous EVAS procedure using the Nellix system (Endologix Inc.) carried at another hospital 17 months before.

**Fig. 1:**
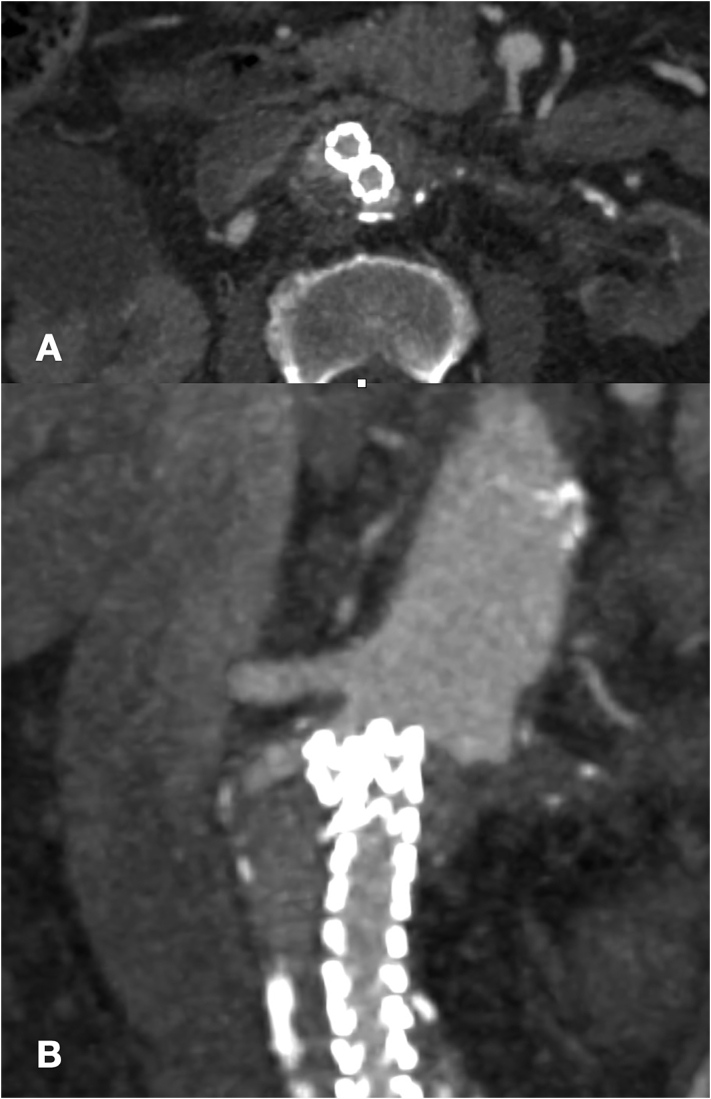
Preoperative CT Angiography showing endoleak in traversal plane (A) and coronal plane (B).

**Fig. 2:**
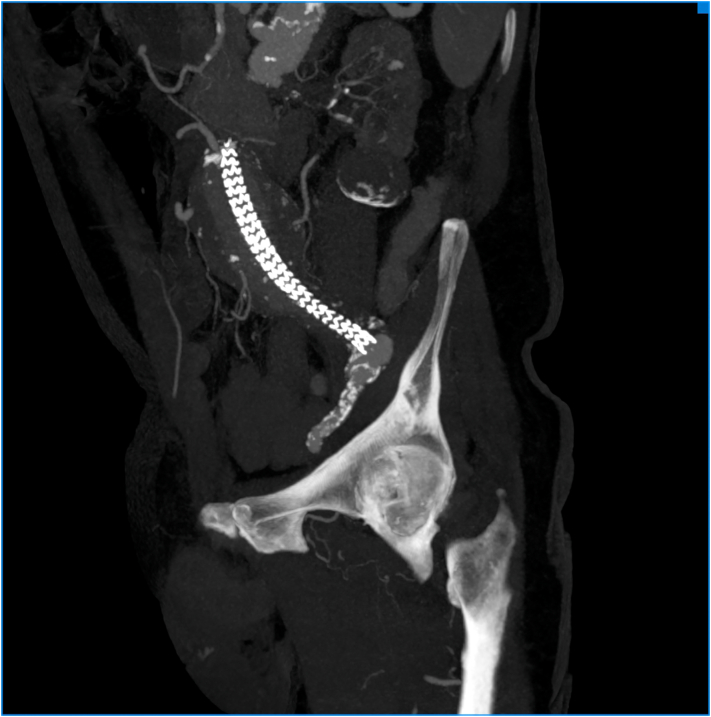
Preoperative CT Angiography showing left iliac aneurysm.

The angio-CT scan confirmed the previous EVAS procedure and showed a type Is2 endoleak below the right renal artery resulting in a 2.5 cm aortic blister and contrast medium filling the space between the aortic wall and the endobags (Fig. 1). In addition, a 26 mm maximal diameter left internal iliac artery and an occluded left renal artery were evident (Fig. 2).

The patient was considered unfit for conventional open surgery and an endovascular approach was chosen. Different endovascular options have been employed to address such complication, but considering the narrow type 1A endoleak entry a coil embolization was chosen.

**Fig. 3:**
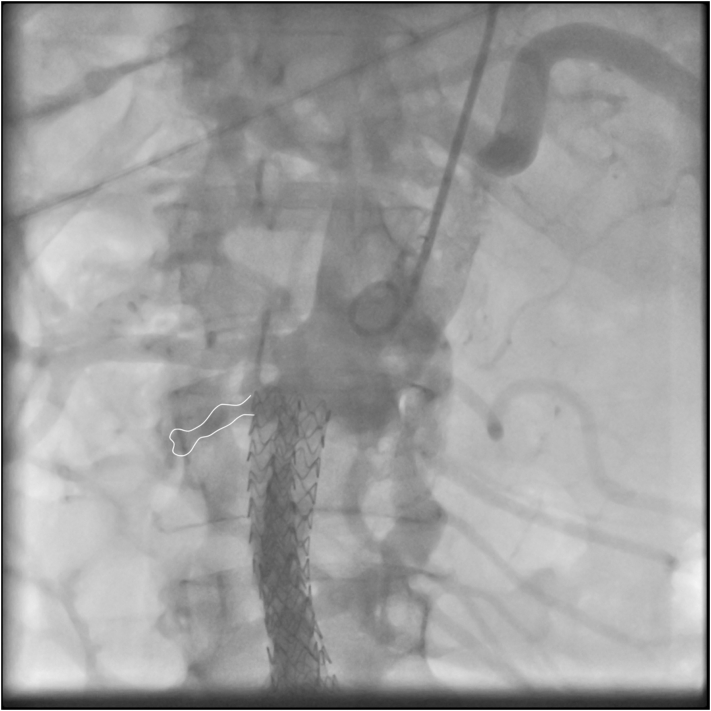
Intraoperative Angiography showing endoleak.

Under local anesthesia, a right radial access was gained and a 6Fx90cm Destination sheath (Terumo Europe, Leuven, Belgium) placed. The sheath tip was placed a few centimeters below the top of the endograft. Anteroposterior and lateral aortograms were performed using a flush catheter paced above the proximal end of the endografts to identify the type 1A endoleak origin (Fig. 3).

**Fig. 4:**
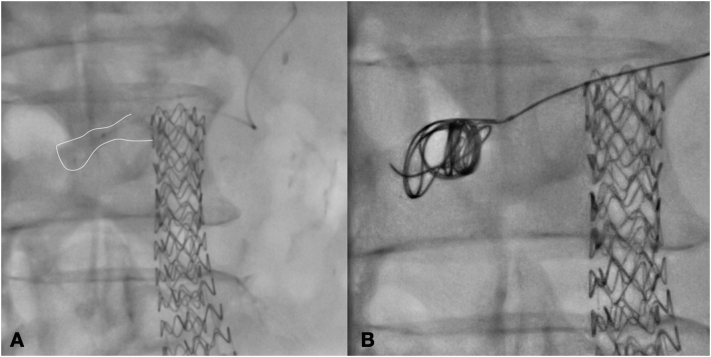
Intraoperative Angiography showing 0.018 microcatheter Rebar18 into the type 1A endoleak cavity(A) and during embolization (B).

**Fig. 5:**
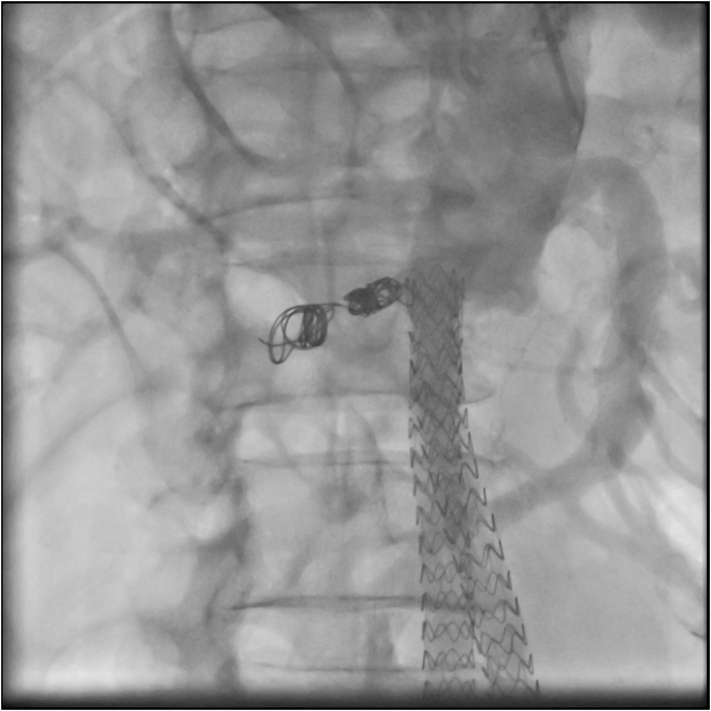
Control angiography showing exclusion of the type 1A endoleak after coil embolization.

The type 1A endoleak entrance was catheterized using a 5F Ber catheter (Cordis, Miami, FL) and a 0.018 microcatheter (Rebar18; Medtronic, Inc., Minneapolis, MN, US) was advanced coaxially under fluoroscopic visualization into the type 1A endoleak cavity. Subsequently through the 0.018 microcatheter, two (16 mm 40 cm first and 14 mm 30 cm second) Concerto Helix Detachable Coil System (Medtronic, Inc., Minneapolis, MN, US) were released inside the aneurysm sac to fill the type 1A endoleak (Fig. 4). The control angiography confirmed the type 1A endoleak sealing after coil embolization (Fig. 5). At 6 h from the intervention the patient was asymptomatic for abdominal pain and stable laboratory tests; the patient was discharged on the 3rd postoperative day.

**Fig. 6:**
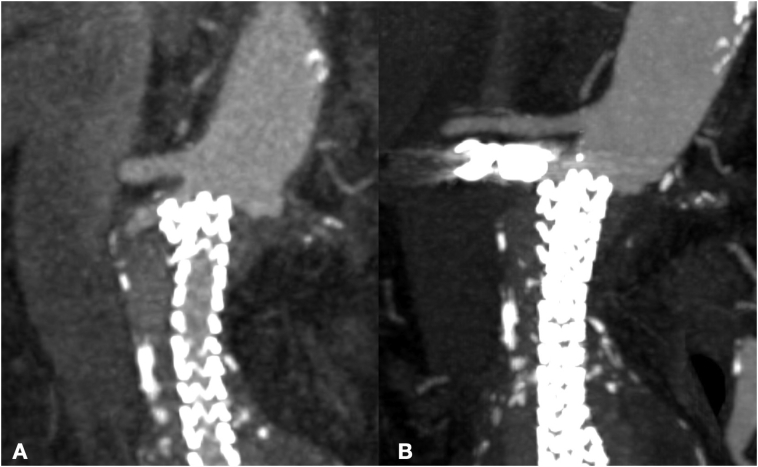
Preoperative CT Angiography with endoleak (A) and 6-months postoperative CT Angiography (B).

The 12 months CT-angiography showed complete type 1A endoleak sealing and Nellix system (Endologix Inc.) stability (Fig. 6).

## 3 Discussion

Endoleaks are EVAR-related complications occurring immediately or during the follow-up [4]. One of the potential advantages introduced with EVAS was a reduced endoleak occurrence [5].

Despite this assumption, when compared to standard EVAR, EVAS was associated with a higher endoleak occurrence and device failure up 43.5% at long-term [2]. Actually, all patients treated with Nellix system (Endologix Inc.) are at risk of AAA growth and stent migration independently forms instruction for use adherence [6].

A different concern regarding type IA endoleaks was reported after EVAS due to a different aneurysm exclusion. The different EVAS implantation rationale required a different endoleak classification as proposed by van den Ham et al. with the aim to anticipate progression and the eventual treatment. Type Is1 endoleak was defined as contrast passage between the endobag and the wall of the proximal neck, not reaching the aneurysm sac itself; Type Is2 endoleak as contrast passage between the endobag and aneurysmal wall or thrombus inside the aneurysm sac; Type Is3 as contrast or fresh thrombus between the endobags inside the aneurysm sac; and Type Is4 endoleak as aneurysm sac pressurization without an evident contrast presence into the excluded aneurysm [7].

In addition, the Nellix endobags filled with the polymer present different density at different interval time after implantation, making endoleak diagnosis complex especially early after implantation [8].

Type I endoleak occurrence after EVAR is a clear indication for reintervention [9]. Different approaches have been proposed including the Nellix system (Endologix Inc.) surgical explanation; Nellix in Nellix application (NINA); proximal embolization. Singh et al. reported a large experience in EVAS failure management with a clear indication to surgical explanation in fit patients. The same authors argued that NINA could not represent an option due to the resilience from the manufacture to provide graft removed from the market [2]. However, it can be argued that all the worldwide implanted Nellix system (Endologix Inc.) would require a reintervention for device failure, and most of them will be unfit for conventional surgery, as reported in our case.

In these circumstances, for asymptomatic patients observation could be an option; for symptomatic and/or ruptured cases alternative therapeutic options has to be considered.

Proximal embolization has been associated with high failure incidence and inefficiency regarding Nellix system (Endologix Inc.) migration [2]. In this single case experience the 6-months CT angiography showed complete AAA exclusion and Nellix system (Endologix Inc.) stability.

## 4 Conclusion

Late failures after Nellix system (Endologix Inc.) are supposed to be observed due to graft failure. Patients requiring emergent treatment for type I endoleak should be managed by surgical Nellix system (Endologix Inc.) explanation. NINA in unfit patients for open surgery patients is excluded due to manufacture restriction with the Nellix system (Endologix Inc.). Despite concern in term of durability, proximal embolization is feasible and this tool should be taken in account especially in emergent setting when no other surgical options exist.

## Funding

None.

## Ethical approval

None

## Consent

Written informed consent was obtained from the patient for publication of this case report and accompanying images. A copy of the written consent is available for review by the Editor-in-Chief of this journal on request.

## Author contribution

Ettore Dinoto: study concept, design, data collection, data analysis, interpretation, writing the paper, final approval of the version to be submitted, **guarantor**.

Felice Pecoraro: study concept, design, data collection, data analysis, interpretation, writing the paper, final approval of the version to be submitted.

Francesca Ferlito: study concept, design, data collection, data analysis, interpretation, final approval of the version to be submitted.

Graziella Tortomasi: study concept, design, data collection, final approval of the version to be submitted.

Domenico Mirabella: study concept, design, data collection, final approval of the version to be submitted.

Guido Bajardi: study concept, design, data collection, data analysis, interpretation, final approval of the version to be submitted.

## Guarantor

Ettore Dinoto.

## Provenance and peer review

Not commissioned, externally peer-reviewed.

## Declaration of competing interest

The authors have no ethical conflicts to disclose.
